# Development, implementation and early results of a 12‐week web‐based intervention targeting 51 children age 5–13 years and their families

**DOI:** 10.1002/osp4.440

**Published:** 2020-07-15

**Authors:** Annelie Thorén, Annika Janson, Erling Englund, Sven‐Arne Silfverdal

**Affiliations:** ^1^ Department of Clinical Sciences, Pediatrics Umeå University Umeå Sweden; ^2^ Department of Pediatrics Sollefteå Hospital Sollefteå Sweden; ^3^ National Childhood Obesity Centre Karolinska University Hospital Stockholm Sweden; ^4^ Division of Pediatric Endocrinology, Department of Women's and Children's Health Karolinska Institutet Stockholm Sweden; ^5^ Department of Research and Development Västernorrland County Council Sundsvall Sweden

**Keywords:** children, intervention, obesity, web‐based treatment

## Abstract

**Background:**

Internet‐based treatments have proven effective for various health issues. There is a need to scale up interventions targeting children with obesity, also in less densely populated areas where the prevalence in many countries is higher than in urban areas. The aim of this study was to design and implement an internet‐based program as an add‐on to standard treatment for childhood obesity.

**Methods:**

Web‐Childhood Obesity Prevention (Web‐COP) was a prospective feasibility study with a pre‐ post‐ design. The intervention consisted of four group‐based education sessions at the clinic, physical activity on prescription, and a new 12‐week internet‐based program. Web‐COP was offered to children with obesity (International Obesity Task Force Body Mass Index (IOTF‐BMI) ≥ 30 kg/m2) and their parents in two counties in Northern Sweden from August 2018 to June 2019. The primary outcome was change in BMI standard deviation score (BMI‐SDS).

**Results:**

The study included 55 children 5–13 years of age. The internet‐based component was well received, and retention rate was 51/55 (92.7%). Data was analysed for 51 children. Mean BMI‐SDS was 3.3 at start and decreased by 0.2, 0.3, and 0.4 at two, four, and six months from baseline. Using a continuous algorithm, 42/51 (81%), children lowered their BMI‐SDS and 33/51 (65%) lowered their BMI.

**Conclusion:**

Adding group sessions and an internet‐based program to standard care was feasible and two thirds of included children with obesity reduced their BMI.

**Abbreviations**
BMI: Body Mass Index
IOTF: International Obesity Task Force
Web‐COP: Web‐Childhood Obesity Prevention

## 1 INTRODUCTION

Childhood obesity is a growing global health concern.^1^ Obesity in childhood is likely to track into adulthood.^2^, ^3^, ^4^ Longitudinal data from children born 1997–1999 in Sweden show that children with overweight or obesity at five years of age tend to remain living with overweight or obesity later in childhood.^5^ However, there is also considerable new development of obesity.^2^, ^4^, ^6^ Early interventions targeting children with obesity 5–13 years of age serve as attractive opportunities for promoting a healthy lifestyle aiming at reducing future risk of obesity and associated health risks.

In data from Sweden's three largest cities, 9–16% of four‐year‐old children have overweight, and another 2–4% have obesity.^7^, ^8^, ^9^ The previous rapid increase in the prevalence of childhood obesity in Sweden may have haltered.^10^ The prevalence of childhood and adult obesity is higher in the less densely populated northern parts of Sweden than in other parts of the country.^11^, ^12^ A decline in childhood overweight and obesity has been noticed also in rural areas. In a study on four‐year‐old children living in Northern Sweden the prevalence of overweight among boys fell in five years from 17.2% to 14.2% and among girls from 22.3% to 19.0%.^13^

Treatments of childhood obesity emphasize lifestyle modification, that is, restrict calorie intake and increase energy expenditure by increased physical activity. Evidence suggests that multi‐component interventions that aim at stimulating changes in behaviour may achieve small reductions in body weight status in children.^14^, ^15^, ^16^ Also, previous studies have shown that involving parents is beneficial in child obesity treatment.^17^, ^18^, ^19^, ^20^, ^21^ The benefit of parental involvement is likely multi‐factorial, from being a role model and provide psychological support to the practical parental support that is pivotal for children to change dietary habits by providing healthy meals and limit exposure to unhealthy foods, promote physical exercise, and be able to travel and join treatment programs.^13^, ^22^ Many previous trials on childhood obesity treatment report high levels of drop‐out or loss to follow‐up, with drop‐out rates ranging from 40% to 79%.^13^, ^16^, ^22^ In less densely populated areas, the distance to treatment centers may further limit the ability for children to participate in obesity treatment programs.

Web‐based treatments in various health‐related areas have demonstrated to be effective with low risks. Using the internet for communication with patients has promoted patient satisfaction, coverage of treatment, and cost‐effectiveness.^23^ Weight loss programs delivered over the internet could be especially appealing to children and adolescents.^24^ Inherent attributes of web‐based interventions, such as interactivity, flexibility in time and location, and anonymity, which may limit obstacles due to stigma related to obesity, may be beneficial.^24^, ^25^ Still, there is a need to verify that web‐based interventions help to sustain long‐term behavioural change. In a review of twelve studies describing eight randomized web‐based interventions, four of the studies showed a reduction of obesity in the different measurements of obesity used, whereas two studies showed no results. In two studies, the BMI increased at follow‐up.^26^ More than a decade ago, a randomized controlled study of a comprehensive one‐year intervention promoting lifestyle changes in overweight and obese children age 8–12 years in Northern Sweden showed modest effects,^16^ but, interestingly, the study had an internet‐based follow‐up during the second year. The authors concluded that families were not comfortable with the internet‐based part of the program and the platform was not much used.^27^

In the area of this study, standard care treatment for children with obesity comprises a doctor's appointment and, in some cases, contact with a dietician. This study, the Web‐Childhood Obesity Prevention (Web‐COP) study, offered an add‐on to existing standard care. The study aimed at designing a web‐based module and investigating whether the addition of a web‐based module was feasible from a technical and practical point before planning for a randomized controlled study. More specifically, the study wanted to asses if the various means of recruitment of patients would work, if there were technical issues on the use of the internet‐based modules, and if internet based weekly modules could assist by supporting other parts of the program and increase the participation of the families, limit drop‐outs and increase the frequency of treatment contacts, without the family needing to attend the clinic. The hypothesis was that school nurses would be able to recruit patients that had not previously been in treatment for obesity, that the parents and families would use and find the new internet‐based program attractive, that children would remain in treatment. This paper aims to present the design and feasibility aspects of patient recruitment, internet‐based activities, and retention rate, as well as, changes in BMI‐SDS at two, four, and six months after inclusion.

## 2 MATERIALS AND METHODS

### 2.1 Study area, recruitment, and study population

The study took place in Västernorrland Region in the north of Sweden. In this area, 98% of the population has access to high‐speed internet.^28^ Families from both urban and rural areas, including parents living in separate homes, were invited to participate in the study if interested and eligible. Participants were recruited in cycles 2018/2019, with two groups starting the program in August 2018 and one group starting in January 2019. A variety of recruitment strategies were utilized, including newspapers, study pamphlets, and direct contact (AT) with school nurses. Children and parents were invited to the paediatric health care clinics at the hospitals in Sollefteå and Sundsvall for a baseline visit and measures of weight, height, and waist circumference prior to the first group session.

Inclusion criteria were obesity as defined by the International Obesity Task Force (IOTF) cutoff IOTF‐BMI ≥ 30,^29^, ^30^ 5–13 years of age, families able to speak and write Swedish, and having a high‐speed internet connection in their homes. Previous obesity treatment was not an exclusion criterion.^25^ Syndromic obesity, or a major neurological or endocrine disease, were exclusion criteria. A modification of the protocol extending the upper age limit to 13 years was made to enable five children age 13 years to participate.

### 2.2 Study design

**FIGURE 1:**
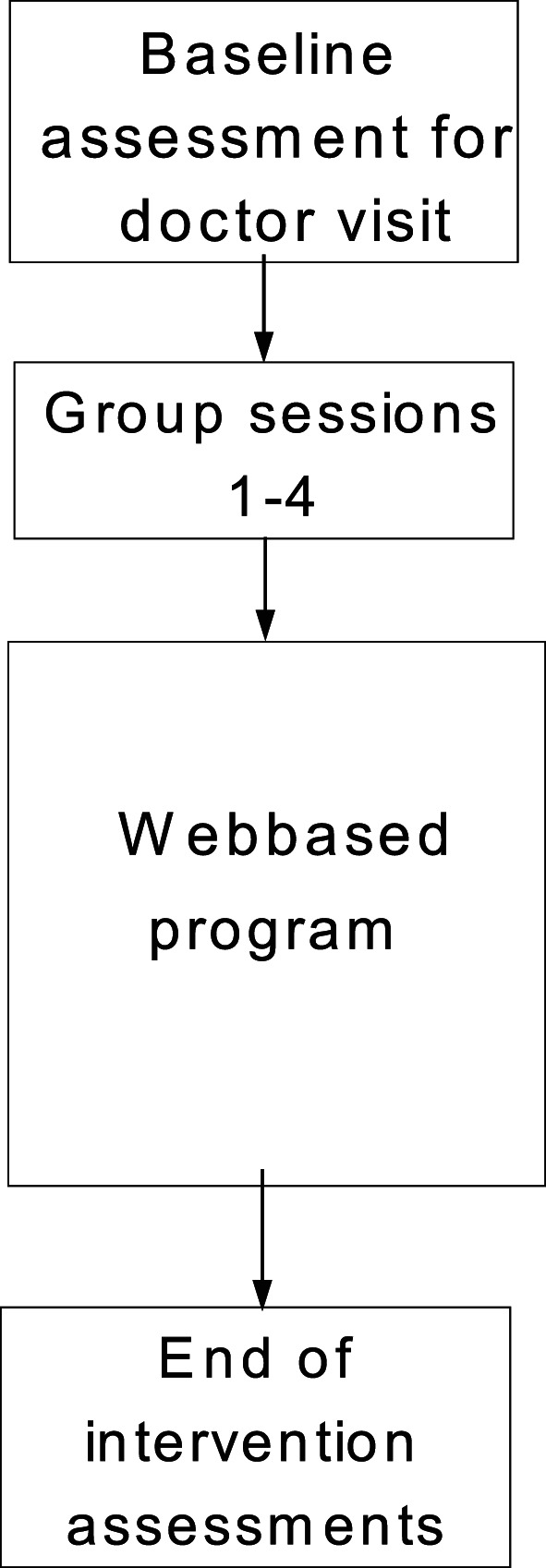
The timeline of the Web‐COP program

This was a two‐center, single‐group, non‐randomized, prospective feasibility study with a pre‐test and post‐test design aimed at evaluating the feasibility of the new intervention, including educational group sessions, and a web‐based treatment program. The Web‐COP study consisted of three components: 1) a series of four educational group sessions, 2) a 12‐week web‐based program, including coaching, and 3) physical activity on prescription^31^ (Figure 1).

### 2.3 Group sessions

After the initial inclusion visit to the paediatrician, group sessions were given weekly over a four‐week‐period to parents and children. The group sessions took place at an exercise center in each community. Each session lasted 120 minutes and was attended by the child and at least one parent. Parents and children met in separate groups for physical activity and nutrition lessons. At all sessions, the children engaged in physical exercise for 45–60 min. Children were given the opportunity to try various sports like outdoor training, swimming, Tabata, and circuit training. Meanwhile, parents were given education in nutrition and physical activity.

Two experienced members of staff, a dietician and a physical therapist, led the classes and encouraged discussions on different topics. Lessons on nutrition focused on the importance of increasing fruit and vegetables, avoiding food high in sugar, and introducing *my plate* (Tallriksmodellen) to focus both on meal size and meal composition. These lessons were provided to children through interactive activities. Parents and children also had the opportunity to prepare one simple healthy meal together, and parents were offered a tour with a dietician in a grocery store to learn more about healthy and unhealthy food choices. Between the meetings, children and parents had home assignments related to the theme of the meeting. In one group session, the parents were provided instructions on how to use the internet‐based program and were given technical support. The outline of the internet‐based program content was delivered verbally to the group supplemented with a slide presentation. Printed material was provided to the parents during two sessions.

### 2.4 Internet‐based program

The mobile‐phone friendly internet‐based program was designed for parents to support their children's obesity treatment. The internet‐based program was developed by a multidisciplinary team of researchers and experts in nutrition, paediatric medicine, engineering, child psychology, web‐design, and physical activity. Gamification, application of game‐design elements, was used to support families in achieving positive health behaviour goals. The parents received a personal login to the internet‐based program. For 12 weeks, the parents participated in a program consisting of online lessons with different topics. Parents could complete the program at their own pace. Each week ended with a test for the participating parent or family. After finishing each week, the parents received a short message to praise the parent for a completed week and to encourage them to start the next week in the program.

The twelve sessions aimed at encouraging healthy eating habits, such as choice of breakfast and reducing sugar intake, and gave examples on how to increase the amount of fruit and vegetables when planning a meal. In addition, the sessions aimed at motivating the family to be more physically active. The program also contained quizzes on healthy food choices and information about how to promote healthy sleeping and screen‐time behaviour. If parents had not used the internet‐based program for a couple of days, they received a reminder message via mail. Both parents were asked to participate in the web‐based program.

### 2.5 Physical activity on prescription

At the inclusion visit, the paediatrician discussed the importance of getting 60 minutes or more of physical activity per day with the children and parents and prescribed “physical activity on prescription”. Physical activity on prescription is a Swedish structured national model that enables health care providers form different cadres to prescribe physical activity.^32^ The prescription enabled the children to get free access to activities from a local provider of the family's choice. The younger children could choose between different physical activities, including family aerobics and swimming, while the older could choose spinning, gym weight training, aerobic circuit training, or swimming. Parents were encouraged to exercise with their children and to promote more daily activities together and time outdoors.

## 3 ETHICS

Children and families were invited to participate and received information, both verbal and written. Verbal informed consent was received for the baseline measurements and written informed consent from both child and parents was received at the baseline doctor's appointment. The study was approved by the Ethics Board at Umeå University (Dnr 2018–113‐31 M, Dnr 2018–461‐32 M).

## 4 DATA COLLECTION

Patient files at the clinic were used to determine if patients were previously treated for obesity. Patients who had no previous contact recorded for obesity were defined as “new” patients. Attendance was taken at all group sessions. The registration to use the internet‐based program was recorded for each family, and weekly use was reported by the parents.

### 4.1 Measurements

BMI was calculated by weight (kg) divided by height squared (m^2^) and using growth references of the Swedish growth charts for numerical value of BMI‐SDS.^33^ Children's heights and weights were measured at the start of the intervention (baseline) and after two, four, and six months. Height was measured to the nearest 0.1 cm. Weight was measured with the children barefoot in light clothing the nearest 0.1 kg using a digital scale (Tanita Digital Scale). Data was collected in Sollefteå and Sundsvall separately, and the same paediatric nurse made all measurements of children in each clinic. Data were entered in the Swedish Childhood Obesity Treatment Register (BORIS), a national quality register supervised by the National Board of Health and Welfare in Sweden.

### 4.2 Statistical analysis

As data on BMI‐SDS were skewed and not normally distributed, data were log transformed. Standard descriptive statistics (geometric mean, as the analysis was based on log‐transformed values and 95% confidence interval) were computed using the continuous algorithm for BMI‐SDS using the Swedish growth references and growth charts.^33^ A paired two‐tailed t‐test was used for significance testing with the geometric mean of the ratio of paired values as measures of difference with a 95% confidence interval. The significance level was set at *p* < 0.05 (IBM SPSS Statistics, Version 25.0, IBM Corp, Armonk, NY, USA) was used for the analyses. Seven children have a missing value at one of the measure points. In these cases, imputations with *Last observation carried forward* (and similar if an observation was carried backward) or means of nearby values, was used. Of these, one child has a missing BMI‐SDS at baseline, and it was replaced by the value at two months. Two children had a missing BMI‐SDS at four months and it was replaced by the mean of two and six months. Four children had a missing BMI‐SDS at six months, and it was replaced by the value at four months.

## 5 RESULTS

A total of 59 children (31 girls) and their parents were recruited in two communities. Recruitment was primarily done by school nurses, but also children already in treatment at the paediatric clinic were asked if they would be interested in participating. Of included children, 28 (16 girls) had previously not been attending obesity treatment at the paediatric clinic or elsewhere. Since many persons were involved in recruiting, we were unable to determine how many families were informed about the project.

**FIGURE 2:**
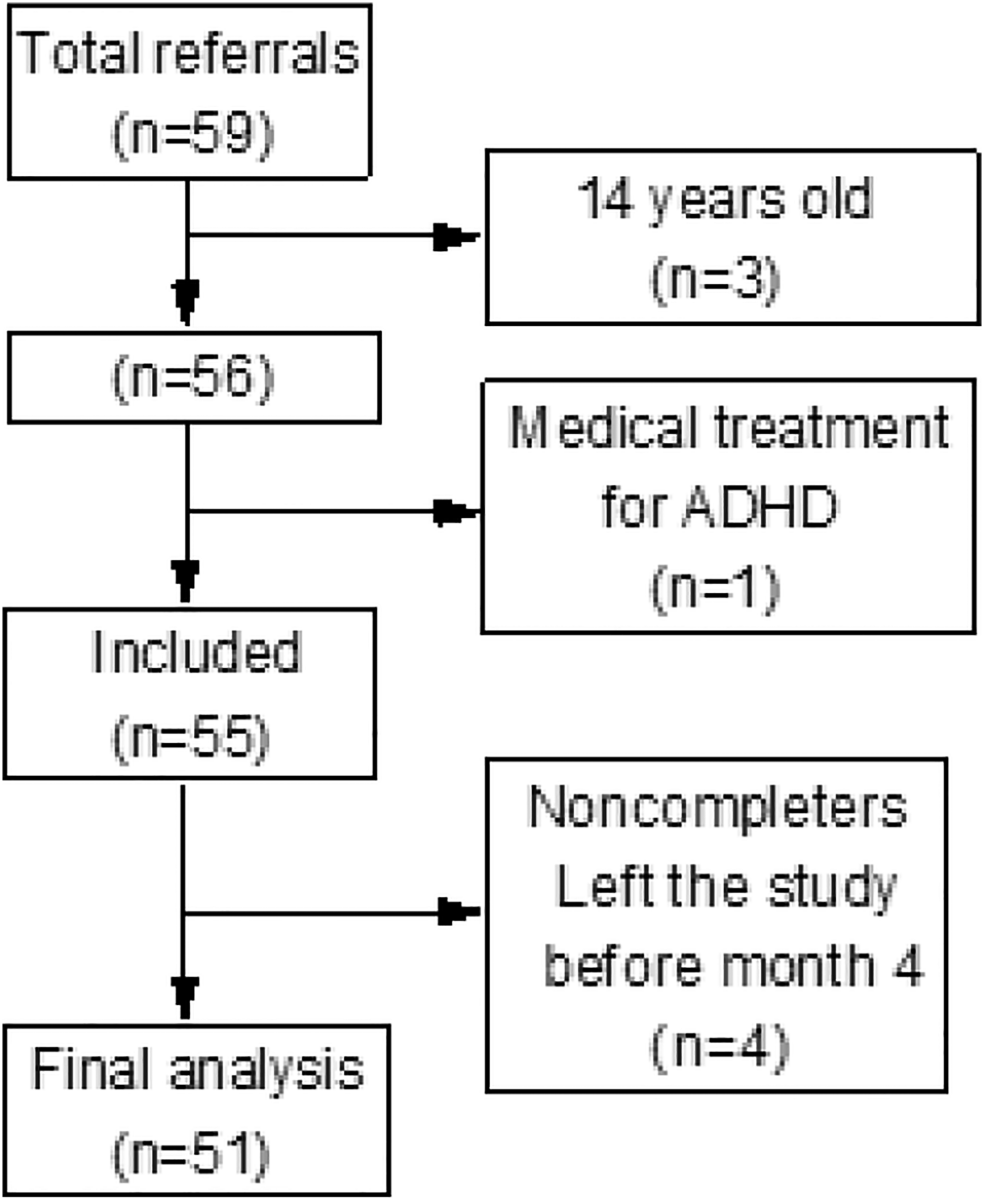
Web‐COP CONSORT

Three children were excluded because they passed 13.9 years of age at the start of the intervention, and one participant was excluded because of taking medical treatment for ADHD. Four left the study before four months. In total, 51/55 (92.7%) of included children (27 girls) aged 5–13 completed the intervention (Figure 2).

In 28 (54%) of 51 included children, at least one parent and child participated in all the four educational group sessions. At least one parent to each child registered in the web‐based program and most parents were also active in the internet‐based program. The four families that withdrew did so in the period of the group sessions, and their rationale for not continuing was that they had a long way to drive to the group sessions and did not see obesity in the child as a big problem. The remaining 51 children and their parents completed the group sessions and proceeded to support through the web‐based program. All families completed the web‐based part of the program.

**FIGURE 3:**
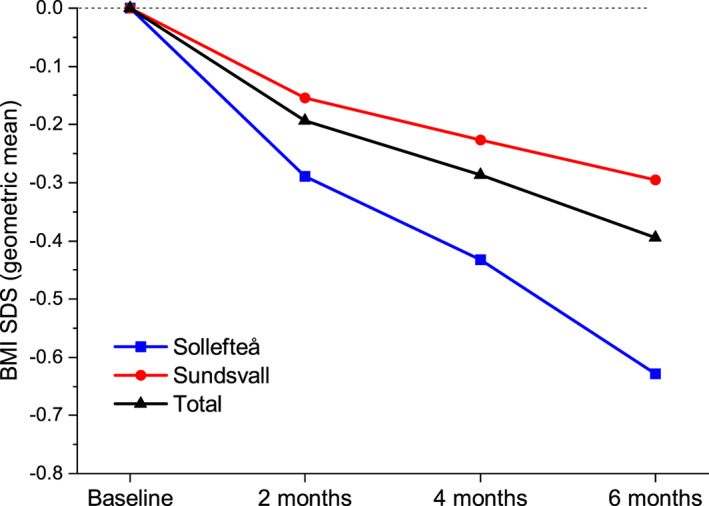
Web‐COP BMI‐SDS

Of the 51 children, 42 lowered their BMI‐SDS during the six‐month intervention. Four children had a higher BMI‐SDS after the intervention, and data at six months are missing for four children. The mean BMI‐SDS was reduced from 3.3 (95% CI: 3.0–3.6) at baseline to 2.9 (95% CI:2.7–3.1) (geometric) measured at six months (*P* < 0.001), the ratio of six months and baseline: 0,88 (95% CI: 0.85–0.91), meaning that BMI SDS has decreased 12% since baseline. A decrease in BMI‐SDS was shown already after two months (0.94 (95% CI: 0.92–0.96), P < 0.001). The children in Sollefteå had higher BMI‐SDS at baseline compared with the children in Sundsvall, 3.5 (95% CI: 2.9–4.3) 3.2 (3.0–3.5), respectively. The reduction in BMI‐SDS was greater for the children in Sollefteå. The reduction in both Sundsvall and Sollefteå were significant (*p* < 0.001, Sundsvall: 0.91 (95% CI: 0.88–0.94) and Sollefteå: 0.82 (95% CI:0.77–0.88)) (Figure 3).

## 6 DISCUSSION

This study, the Web‐COP program, was developed as an add‐on to standard care and aimed at increasing parents' awareness, motivation, and ability to modify habits affecting their children's weight and health. The three components of the program were meant to reinforce each other: group sessions, a web‐based program, and organized physical activity free of charge. This was a feasibility study^34^ with a focus on the study process, and we conclude that the recruitment process worked and that school nurses were effectively informing families about the study. The sex distribution of the included patients was balanced. The drop‐outs occurred during the period of group sessions only, and all remaining families were participating in the internet‐based program. There were no major technical issues, and we observed no negative side‐effects of the program. Data on changes in BMI‐SDS were quite promising in relation to other treatments of childhood obesity.^14^, ^15^, ^35^, ^36^, ^37^

With the high prevalence of childhood obesity there is a need to scale up interventions that are easily accessible and appreciated as helpful by the parents. This study included 24 (47%) children that were not previously in treatment. Also, the attrition rate (92.7%) was higher than in many programs targeting childhood obesity,^1^ likely beneficial for the outcome in terms of BMI‐SDS. With the aid of the child's parents, interventions should aim at diminishing the risks of persisting obesity and associated risks. Previous studies support the programs that target parents to children with obesity,^19^, ^20^, ^21^ but results on web‐based treatments for treating childhood obesity were inconclusive. The authors of the review of twelve studies on using internet in obesity treatment concluded that controlled studies on stand‐alone web‐based treatments might elucidate their usefulness.^26^ In contrast, our design was based on a presumption that web‐based components would be useful as one of several components in an intervention. In the previously mentioned comprehensive one‐year randomized controlled study aimed at promoting lifestyle changes in overweight and obese children age 8–12 years in Northern Sweden 2006–2009, the authors reported that the web‐based support in the second year was not much used by the families and the drop‐out rate of the total study was 45%.^16^, ^27^, ^38^ In our view, the increased usage of all kinds of internet applications over the last decade has largely contributed to the higher acceptability of the web‐based component in this study, and likely also to the attrition rate.^26^

The prevalence of overweight in children and their parents has shown to be higher in rural compared with urban areas.^12^, ^39^ Moraeus et al. have suggested that targeting rural areas might be an effective approach to reach children with the greatest needs.^39^

Two‐thirds of the children in this study reduced their degree of obesity. The difference in results between the two sites was most likely due to higher BMI‐SDS at baseline in some children in Sollefteå compared with the children in Sundsvall. Previous studies have shown that even moderate changes in BMI among children can reduce risk indicators for cardiovascular disease.^40^

Our findings suggest that future childhood obesity interventions may benefit from having components like Web‐COP with group sessions supported by a web‐based program. The inclusion of a mobile‐friendly web‐based program is consistent with recommendations of multi‐component interventions to address the complexity of childhood obesity. Based on this feasibility study, further development of the Web‐COP project will include more interactive game‐like activities, less text in the web‐based information, additional group sessions, and the children will be offered a smart‐watch to track daily activity. To further study the effect of Web‐COP, a randomized controlled trial with cross‐over design on a larger number of children for a longer period of time was started in August 2019.

The strength of this study is that about 90% of the included patients completed the study, which is a high attrition rate for interventions on obesity. A limitation of the design was that families left the study during the period of group sessions, whereas they could have benefitted from the web‐based intervention that came after the group‐sessions. This will be considered for future studies. The major limitation of this study was its lack of randomization and control group.^41^ Also, we cannot determine which components of Web‐COP were useful, just that the whole intervention achieved these results. Another limitation is the short‐term follow‐up. Also, the study could be subject to confounding factors due to selection bias as families were recruited for their willingness to participate. Since many persons were involved in the recruitment and the study was presented in schools and media, it was unforunately impossible to determine how many families were introduced to the study and asked whether they wanted to participate. Recruiting patients based on their willingness to participate is a built‐in limitation to the design of this study aimed at encouraging reflecting on habits, and sustainable behavioural change, which can only be achieved with the active involvement of the patients. More studies are necessary to confirm the evidence of combined group sessions and web‐based family interventions.

## 7 CONCLUSION

The Web‐COP program with group sessions, an internet‐based program, and physical activity free of charge was feasible in terms of patient recruitment and attrition, and the web‐based component was well accepted and used by the families. The results on changes in BMI‐SDS were promising and 42 of the 51 children lowered their BMI‐SDS, and the average reduction was 0.4 SD during the six‐month intervention. Even a small reduction in mean BMI‐SDS is beneficial to the child's health. To confirm the findings of this feasibility study, a randomized controlled trial with a longer follow up is now underway.
